# Long-term quality of life and functional outcomes in adults surgically treated for intramedullary spinal cord tumor

**DOI:** 10.3389/fpsyg.2023.1136223

**Published:** 2023-04-20

**Authors:** Tommaso Tufo, Eleonora Grande, Giuseppina Bevacqua, Ines Di Muccio, Beatrice Cioni, Mario Meglio, Marco Ciavarro

**Affiliations:** ^1^Department of Neuroscience, Neurosurgery Institute, Policlinico A. Gemelli Foundation University Hospital I.R.C.C.S., Catholic University of the Sacred Heart, Rome, Italy; ^2^Neurosurgery Unit, Fakeeh University Hospital, Dubai, United Arab Emirates; ^3^Department of Neurosurgery, S. Anna University Hospital, Ferrara, Italy; ^4^Department of Neurosurgery, AORN Sant'Anna e San Sebastiano, Caserta, Italy; ^5^Section of Neurosurgery, Department of Neurosciences Biomedicine and Movement Sciences, University Hospital, Verona, Italy; ^6^I.R.C.C.S. Neuromed, Pozzilli, IS, Italy

**Keywords:** quality of life, long-term outcome, intramedullary tumors, psychological assessment, psycho-oncology

## Abstract

**Introduction:**

Intramedullary spinal cord tumors (IMSCTs) are rare but clinically significant entities that may cause severe neurological decline with progressive pain and motor or sensory deterioration. Beyond the beneficial effects of surgical treatment and the long-term progression-free survival, neurological deficits may still persist after surgery, and information about the long-term patients' health-related quality of life (QoL) is still lacking. In this study, we investigate the patients' health perception 15 years after the surgery in an overall patients' wellbeing framework.

**Methods:**

Patients surgically treated for IMSCT over a period from 1996 to 2011 were selected. After a mean of 15 years from the surgery, patient's self-administered questionnaire on disability, pain, sleep quality, and QoL was collected and neurological postoperative evaluation at the chronic stage was reexamined.

**Results:**

Neurological deficits are reported in half of the patients in the postoperative chronic phase. After 15 years of surgery, half of the patients still report mild or severe disability grades associated with significantly higher pain and poor sleep and QoL. In accordance, the neurological condition measured at the chronic stage is significantly related not only to disease-specific symptoms (i.e., pain) but even to sleep quality complaints and poor QoL, measured at 15 years follow-up.

**Conclusions:**

Health-related QoL is an important secondary outcome in patients. Although the progression-free survival, worse postoperative neurological conditions could predict long-term sequelae reflecting patients' poor health perception. It suggests the importance of preserving patients' functional status and globally evaluating patients' wellbeing to handle disease-specific symptoms but even more general aspects of QoL.

## 1. Introduction

Intramedullary spinal cord tumors (IMSCTs) are rare lesions of the central nervous system that involve 5–10% of all spinal tumors in adults (Bansal et al., 2013). Although often histologically benign with progression-free survival (Gomez et al., 2005; Chamberlain and Tredway, 2011), these lesions may induce neurological deterioration in patients, such as axial and neuropathic pain and motor or sensory deficits (Harrop et al., 2009; Samartzis et al., 2015).

For decades, a conservative approach consisting of biopsy combined with radiotherapy has been posed (Manzano et al., 2008), until Epstein and colleagues proposed a more aggressive surgical resection treatment, demonstrating good neurological and functional results in patients (Constantini et al., 2000). Recently, technological advances in neuroimaging, as well as surgical resection employing the microsurgical technique and intraoperative neurophysiological monitoring have become a successful strategy (Klekamp, 2013; Cannizzaro et al., 2019), with a low complication rate (Samartzis et al., 2015). Notwithstanding the acute, surgery-induced perioperative neurological deterioration, nearly half of the patients reported a neurological improvement to the baseline within a few months (Bansal et al., 2013). However, in one-out-of-two patients, new or increased dorsal column dysfunctions were outlined (Manzano et al., 2008). It has been described as the most adverse postoperative morbidity, including symptoms like paresthesia and loss of autonomic function, motor weakness, generalized numbness or painful dysesthesias, and gait dysfunction. Notably, poor research studies investigate whether these symptoms may globally affect the health-related QoL in IMSCT patients. In the majority of the cases, the postoperative functional outcomes are evaluated in terms of tumor-related prognostic factors and neurological dysfunctions (Innocenzi et al., 1997; Raco et al., 2005; Nakamura et al., 2006; Beneš et al., 2009; Eroes et al., 2010; Khalid et al., 2019). To date, data on QoL after spine surgery are lacking, and the current findings are debated: some research studies show that patients' QoL at follow-up is worse than the normal population (Fisher et al., 2005; Melcher et al., 2007; Liljenqvist et al., 2008); others found more favorable results (Kato et al., 2014; Mazel et al., 2014). A recent study showed moderate impairment in the physical and mental components of QoL, which improved 3 months after surgery but does not result in a complete restoration of optimal QoL levels (Luzzati et al., 2020). Clear and reliable data on patients' self-perception of the diseases can be useful to assess these patients' needs in a rehabilitation framework. At the same time, longitudinal inquiries are lacking as well (Luzzati et al., 2020). Although IMSCT is a long-term progression-free survival disease, the neurological and psychological consequences of IMSCT persisting symptoms are recorded until a few months after surgery, usually 12–36 months. It is, therefore, crucial to employ a multidimensional approach in examining patients' outcomes to understand the IMSCT management and the IMSCT impact on the patient's QoL better even long after the surgery.

Our study aimed at investigating the long-term self-perception of health in patients who were surgically treated for IMSCT and whether the neurological status could impact patients' daily activities and health perception long after the surgery. Data on perceived disability, pain, sleep quality, and QoL were collected after a mean of 15 years from the surgery. Thereafter, we retrospectively compare the scales' scores with the postoperative neurological status. Investigating the impact of IMSCT on long-term patients' health-related QoL could help identify those patients at risk of worse outcomes and plan the best strategies for personalized patients' rehabilitation during the postoperative period.

## 2. Materials and methods

A single-center retrospective review of 48 adult patients who consecutively underwent IMSCTs resection with intraoperative monitoring between 1996 and 2011 at the Institute of Neurosurgery of Polyclinic A. Gemelli University Hospital IRCCS of Rome was performed.

Inclusion criteria involved IMSCTs surgical treatment, regardless of the etiology, that was between 10 and 20 years ago; having a minimum of 12 months and maximum of 60 months chronic post-surgery neurological follow-up; and consent to participate in the study. The exclusion criteria applied included lack of clinical information about the tumor, surgery management, and functional outcomes and incomplete questionnaire or duplicated answers.

We collected tumor clinical data such as histology, lesion extension, EOR, and clinical information on patient functional status at preoperative (<1 week before surgery), early postoperative (<1 month after surgery), and a postoperative chronic phase (range 1–5 years) through the Modified McCormick scale (MMCS).

The surgery was carried out using a standard dorsal approach with laminectomy. In those cases, in which a multilevel tumor involvement arose, the laminoplasty approach was used. Allowing for a safer surgical procedure, in the whole sample, the surgery was performed employing somatosensory evoked potentials, motor evoked potentials, and D-wave intraoperative neurophysiologic monitoring (IOM). The cord was incised through a midline or lateral myelotomy that was deepened by dissection with microforceps until the tumor was identified and gently the upper and lower limits of the lesion were exposed.

All procedures performed in the study were in accordance with our department's standards and with the 1964 Helsinki Declaration and its later amendments. All patients gave their consent to be contacted for research purposes at the moment of their hospitalization. Informed consent was electronically obtained from all participants before study inclusion.

### 2.1. Health perception questionnaire

Those patients who underwent IMSCT surgical resection at A. Gemelli Polyclinic Neurosurgery department were contacted and asked to undergo an extensive online survey investigating global wellbeing. Patients who had consented to participate in the survey received the questionnaire by email. The survey was administered through the free open-access GoogleTM Forms (https://www.google.com/forms/about/application).

The survey included a brief description of the study, informed consent approval, and sociodemographic questions. A disability subjective grade was evaluated by means of the Oswestry Disability Index (ODI) (Monticone et al., 2009), which is a self-compiled scale investigating the subjective degree of deficit in daily activities. Patients were asked to classify their pain perception utilizing the Pain Catastrophizing Scale (PCS) (Monticone et al., 2012), which is a patient self-administered scale that quantifies individual pain experience by means of three subscales: helplessness, magnification, and rumination. The Pittsburg Sleep Quality Index (PSQI) (Curcio et al., 2013) was administered to evaluate sleep quality and sleep disturbances through seven dimensions, namely, subjective sleep quality, sleep latency, sleep duration, habitual sleep efficiency, sleep disturbances, use of sleep medication, and daytime dysfunctions. Finally, health-related QoL was examined through the SF-36 (Apolone and Mosconi, 1998), which is a thirty-six items scale assessing the QoL on eight subscales ranging from 0 (poor QoL) to 100 (high QoL): physical functioning, role-physical, bodily pain, general health, vitality, social functioning, role-emotional, and mental health.

### 2.2. Statistical analysis

We conducted statistical analysis on R studio software 1.3.1. Comparison between the rank scale was performed employing the Kruskal–Wallis test. One-way analysis of the variance (ANOVA) was carried out to test discrepancies in the scale score, using the degree of neurological disability (MMCS) and perceived disability (ODI) as between-subject factors. *Post-hoc* analyses were conducted with Tukey's test. To better describe those components that significantly contributed to most of the variability, we carried out a principal component analysis (PCA) on PCS and PSQI scales. The alpha level was set at 0.05 for statistical significance.

## 3. Results

A consecutive series of 48 adult patients (27 F, mean age at the time of the surgery 44.8 ± 15) with intramedullary spinal cord tumor underwent resection with intraoperative monitoring at the Institute of Neurosurgery of Polyclinic A. Gemelli University Hospital IRCCS of Rome. A total of 42 patients met our inclusion criteria. Among them, 19 patients took part in a long-term follow-up questionnaire after a mean period of 15 years (ranging from 10 to 20 years) from the surgery and after a mean of 3.2 years (ranging from 1 to 5 years) from the chronic postoperative neurological evaluation.

### 3.1. Clinical data and neurological evaluations

**Figure 1:**
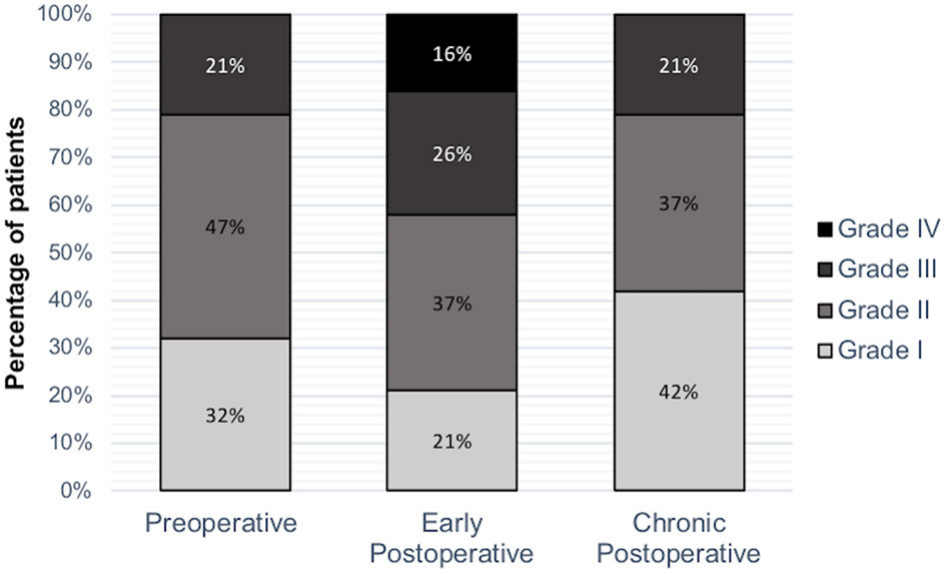
Neurologic evaluation. MCCS value was measured pre and postoperatively and in the chronic postoperative evaluation. A significant difference emerged between early postoperative vs. postoperative chronic stage evaluation (p = 0.01).

A total of 19 patients (11 F) participated in the survey. The sample mean age was 52 years (±11.9). The retrospective evaluation of the clinical data (Table 1) showed 10 patients (53%) with a cervical localization of the lesion, eight (42%) with thoracic one, and one (5%) conus medullaris one. In total, 10 patients (53%) were categorized as having ependymomas, three (16%) with cavernomas, three (16%) with hemangioblastomas, one (5%) with astrocytoma, one (5%) with lipoma, and one (5%) with a dysontogenic cyst. In the great majority of the cases, in 15 patients (78%), the total resection was achieved, while in two patients (11%), there was a subtotal resection, and in two patients (11%), a partial resection. The functional status of patients, evaluated using the MMCS, was 1.8 ± 0.7 at the preoperative stage. It increased to 2.4 ± 1.0 at the early postoperative evaluation and returned to 1.8 ± 0.8 in the postoperative chronic evaluation. No significant difference (*p* > 0.05) was found between pre and early postoperative status, indicating a slight worsening following the surgery. By contrast, a significant difference was registered between the early post-surgery vs. the chronic evaluation (*p* = 0.01), with an improvement of the functional status in the chronic phase (Figure 1). No significant difference emerged comparing the presurgical and the last clinical record (*p* > 0.05), suggesting a recovery to the baseline during the chronic phase.

**Table 1:** Demographic and clinical data.

**Clinical features**						**Survey scale scores**										
						**Disability**	**Pain**	**Sleep**	**SF-36 subscales**							
**Patient**	**Age**	**Gender**	**EOR**	**Istology**	**Localization- extension**	**ODI**	**PCS**	**PSQI**	**Physical functioning**	**Role-physical**	**Bodily pain**	**General health**	**Vitality**	**Social functioning**	**Role-emotional**	**Mental health**
1	59	F	Total	Dysontongenic mass	Cono	0%	0	0	100	100	100	100	100	100	100	100
2	60	M	Total	Ependymoma	Th2-Th6	0%	0	8	100	100	100	65	30	75	100	48
3	41	F	Total	Hemangioblastoma	C2-Th3	0%	0	3	100	100	80	75	65	63	100	80
4	49	M	Total	Ependymoma	Th12-L1	2%	1	1	95	100	87	70	100	88	100	100
5	37	F	Total	Cavernoma	C3-C7	6%	15	8	100	50	65	80	75	63	100	80
6	41	F	Total	Ependymoma	C1-C5	8%	10	2	85	75	74	61	70	75	100	80
7	46	M	Total	Cavernoma	Th10-Th11	10%	5	1	95	100	80	90	65	88	100	92
8	57	F	Total	Ependymoma	C4-C7	11%	10	5	80	100	35	40	55	50	100	52
9	31	F	Partial	Astrocytoma	Th4	16%	24	9	10	50	100	75	65	100	100	92
10	40	F	Total	Hemangioblastoma	C6-C7	22%	8	3	40	50	52	76	45	62	33	44
11	47	M	Total	Ependymoma	Th4-Th5	28%	29	8	55	0	41	25	35	50	0	60
12	64	F	Subtotal	Ependymoma	C1-C3	34%	19	8	70	50	45	40	45	38	33	56
13	49	M	Subtotal	Ependymoma	C4-C6	38%	9	7	20	0	30	61	55	75	66	68
14	72	M	Total	Ependymoma	Th7-Th8	40%	23	8	45	0	22	35	60	88	100	76
15	66	M	Total	Ependymoma	Th8-Th9	50%	36	12	40	0	10	15	30	38	0	16
16	70	F	Partial	Lipoma	C3-C4	51%	19	7	70	0	0	40	40	50	100	76
17	47	F	Total	Ependymoma	Th1-Th3	52%	2	8	35	25	45	35	65	75	67	72
18	57	F	Total	Hemangioblastoma	C2-C5	54%	40	13	30	0	22	20	35	38	0	64
19	65	M	Total	Cavernoma	C4-C6	58%	20	5	5	0	32	15	75	63	0	80
					Mean	25%	14.21	6.11	61.84	47.37	53.76	53.58	58.42	67.08	68.35	70.32
					SD	±0.2	±12.3	±3.7	±33.1	±43.2	±31.6	±26.1	±20.7	±20.1	±42.3	±21.1

Demographic and clinical features of the patients who take part in the survey. Self-administered questionnaire scale scores are reported (mean ± standard deviation).

### 3.2. Health-related follow-up questionnaire

The patients' health-related QoL examination figured out that nine patients (48%) reported a null disability grade on the ODI questionnaire, five (26%) with moderate disability, and five (26%) with severe disability grades. At the PCS scale, two patients (10%) exceeded the cut-off value. The PSQI showed nine patients (50%) reporting normal range scores, whereas the overall wellbeing evaluation of the QoL assessed through the height subscales of the SF-36 questionnaire, is reported in Table 1.

### 3.3. Follow-up questionnaire and postoperative neurological assessment outcome

A series of one-way ANOVAs were carried out to determine whether the degree of disability, both perceived and clinically determined at the chronic postoperative evaluation, was associated with the subjective health-related QoL assessed long after the surgery.

**Figure 2:**
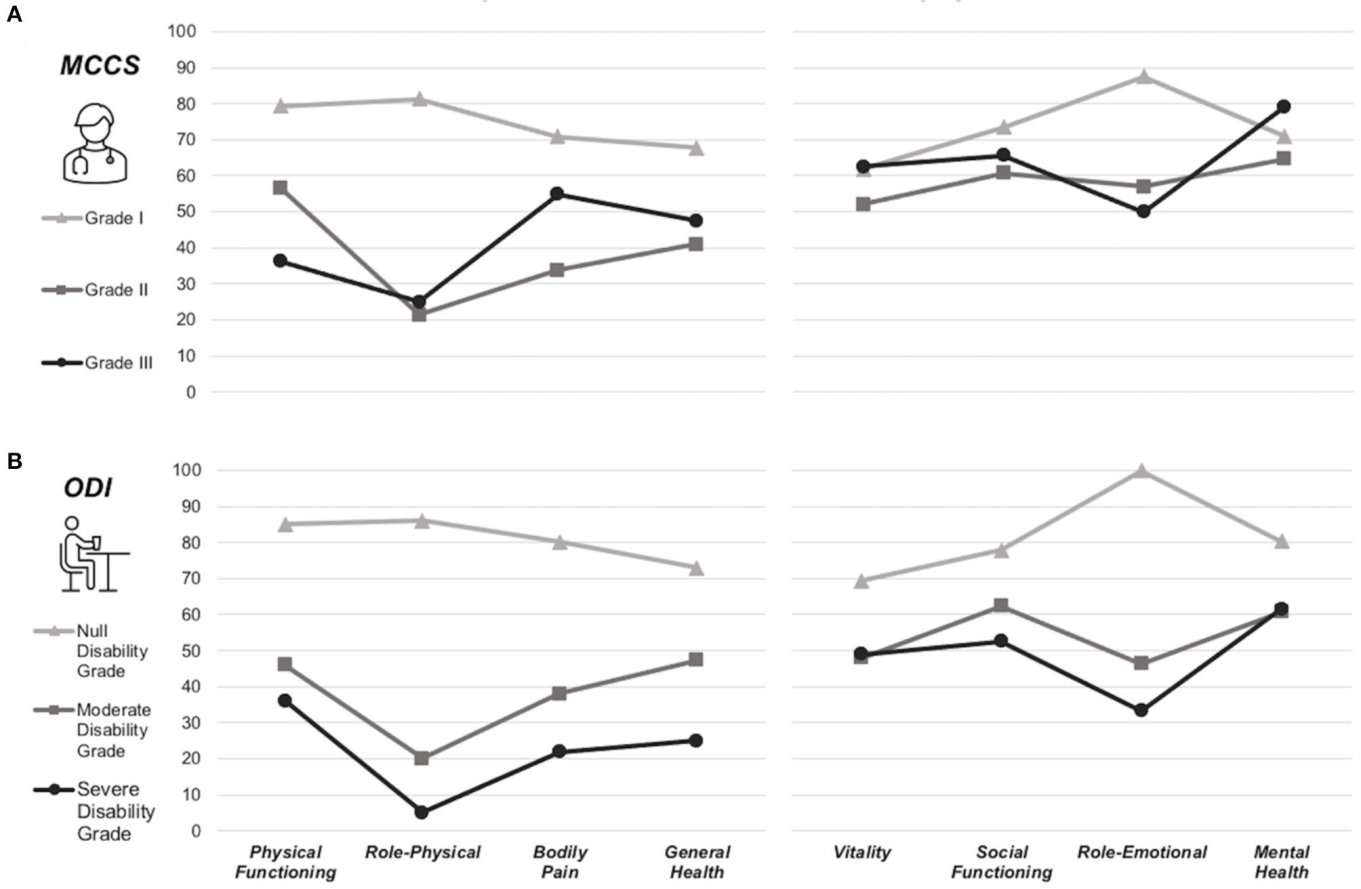
Interaction between SF-36 subscales and the neurological or perceived disability grade. ANOVAs conducted on the SF-36 subscales with **(A)** the degree of clinical disability (MMCS) measured at the chronic stage and **(B)** the perceived disability (ODI) measured at follow-up, as between-subject factors. Different grades of postoperative neurological evaluation reflect differences in the patients' self-perception of health 15 years after the surgery.

The ANOVA performed with the ODI grades of disability (i.e., null, moderate, and severe) as between-subject factors showed a significant interaction among the degree of disability and the PCS and PSQI scales [*F*_(2, 16)_ = 4,067, *p* = 0.037; *F*_(2, 16)_ = 3,963, *p* = 0.039, respectively], with higher perceived pain and poorer sleep quality in those patients reporting severe disability, compared to null disability grade patients. The ANOVA on the eight SF-36 subscales collected in the survey also highlights significant interaction between the subjectively perceived disability grade and the SF-36 subscales score. Notably, the physical functioning [*F*_(2, 16)_ = 324, *p* = 0.005], role-physical [*F*_(2, 16)_ = 28,399, *p* = 0.0001], bodily pain [*F*_(2, 16)_ = 18,781, *p* = 0.0001], general health [*F*_(2, 16)_ = 13, 145, *p* = 0.0004], and role-emotional [*F*_(2, 16)_ = 9,578, *p* = 0.001] significantly differs among the disability grade, with a lower SF-36 subscale score associated with higher perceived disability (Figure 2). *Post-hoc* comparisons revealed significant differences in all the aforementioned scales between the null vs. The moderate grade (*p* < 0.05) and the null vs. The severe grade (*p* < 0.05) of perceived disability. In particular, higher perceived disability results were found to be related to limitations in performing all physical abilities, including self-care (physical functioning); problems with everyday activities due to physical limitation (role-physical); very severe and significantly limiting pain (bodily pain); poor personal health and the belief to health worsening (general health); and problems with everyday activities due to emotional limitation (role-emotional).

Another series of ANOVAs were conducted on self-reported scale scores, considering the MMCS grade (i.e., I, II, and III) measured at the postoperative chronic stage as between-subject factors.

A significant interaction emerged between the MMCS grade and PCS and PSQI scores measured in the survey [*F*_(2, 16)_ = 8.549, *p* = 0.002; *F*_(2, 16)_ = 4.113, *p* = 0.036, respectively], with higher MMCS grade (i.e., poor functional status at the chronic stage) related to higher pain perception and poor sleep quality perception long after the surgery. At the same time, the ANOVA performed on the SF-36 subscales showed a significant interaction with the degree of the role-physical subscale [*F*_(2, 16)_ = 7.174, *p* = 0.005], with lower scale scores associated with MMCS grade II and III (Figure 2). *Post-hoc* comparison showed a significant difference between grade I vs. II (*p* = 0.008) and grade I vs. grade III (*p* = 0.035). Therefore, the role-physical, which specifically reflects the problem in everyday life due to poor accuracy of mobility functions, is still perceived as worse long after the surgery in those patients who showed mild or severe neurological deficits.

Overall, these results indicate that the degree of disability measured in the 15 years follow-up through the self-administered ODI scale or assessed during the postoperative neurological evaluation at the chronic stage using the MMCS scale are both associated with pain perception and sleep quality. In addition, although not all the SF-36 subscales interact with the subjective or objective disability grade, suggesting some scales score may be independent of the functional status, the perceived as well as the neurological disability grade is associated with the health-related QoL, primarily the physical domain, 10 years after the surgery.

Finally, in light of the significative results of PCS and PSQI scales among the ODI and the MMCS score, two principal component analyses (PCA) were performed to investigate which sub-items of PCS and PSQI better explain the total variance. PCA among the seven components of PSQI shows that subjective sleep quality, sleep latency, and sleep duration explain 75% of the total variance. Thus, the reduction of sleep time and, generally, sleep quality were the factors most influencing the reported sleep concerns. The PCA on the three components of PCS indicates helplessness as explaining 84% of the total variance, emerging as the most important driving factor in pain perception, with the weak influence of the rumination and the magnification subscales.

## 4. Discussion

Intramedullary tumors, although benign lesions, when affecting patients' neurological functioning require surgical resection (Persson et al., 2019). Nonetheless, with the recent surgical management with intraoperative neurophysiological techniques, surgery remains challenging, exposing the patients to the risk of neurological deterioration after resection (Samuel et al., 2016; Behmanesh et al., 2020). Acute non-permanent postoperative degenerations are not unusual, with a functional recovery to preoperative status reported in 20–40% of all IMSCT patients only (Samartzis et al., 2015).

Currently, few data are available on patients' self-perception of disability and more general aspects of wellbeing and mental health (Manzano et al., 2008; Guirado et al., 2013; Myrseth et al., 2022). Moreover, although the high survival rate without the progression of the disease, the lack of available results on the long-term functional outcomes after surgical management of IMSCT leads to a limited interpretation of the impact of the disease on the patients' daily activities and QoL. Typically, follow-ups involve evaluation until the last available clinical record, usually 12 months after the surgery (Persson et al., 2019), and in the literature, just a few studies consider postoperative follow-ups longer than 5 years (Robinson et al., 2005; Lee et al., 2014). Moreover, the neurological scales could poorly reflect the global characterization of the patient's condition when a functional deficit occurs (Li et al., 2013; Grande et al., 2022).

To overcome these limitations, the patients' health-related status long after the surgery was evaluated for the first time in this study. To this purpose, after a mean period of 15 years post-surgery, we conducted a survey using self-administered questionnaires investigating patients' QoL, together with a retrospective analysis of the neurological records.

According to the literature (Garcés-Ambrossi et al., 2009), a significant postoperative decline was registered, followed by a functional recovery to the baseline, as indicated by the neurological evaluations at the chronic stage. However, half of the sample still reported mild neurological deterioration. Similarly, the self-administered questionnaires collected 10 years after the last neurological assessment showed poor scores in specific health domains and overall QoL scales.

In particular, half of the sample reported moderate or severe grades of disability on the ODI scale, associated with higher pain perception, poor sleep quality, and life quality (e.g., the belief of health worsening or limitations in everyday activities). More interestingly, even the postoperative chronic neurological evaluation is related to long-term adverse functional outcomes. An association was found between the postoperative MMCS evaluation in the chronic postoperative phase and the overall patient's wellbeing long after the surgery. Hence, higher pain perception, poor sleep quality, and sleep time reduction may reflect the neurological disability grade as well as poor motor function in everyday activities registered 15 years after surgery.

It showed that the MMCS grade measured at the chronic stage might be crucial in estimating patients' status long after the surgery. According to the literature that underlines patients' symptoms, such as weakness and pain, persisting in the postoperative phase (Samuel et al., 2016; Behmanesh et al., 2020), these results showed that the impact of IMSCT surgical resection has long-term outcomes even on functional status. In contrast, those studies showing that most patients reached a good neurological outcome after surgery, even in long-term follow-up (Robinson et al., 2005), may suggest that the routine clinical evaluation might highlight the neurological deficit, not capturing the complexity of patients' health-related condition.

Taking together our results on the association between the chronic postoperative neurological status and the perceived QoL long after the surgery, it could be argued that the importance in the clinical practice is to preserve a good functional/neurological status and to reduce IMSCTs long-term adverse outcomes (Sandalcioglu et al., 2005; Boström et al., 2014), but these findings can be useful even when discussing the patients' surgery consequences, which could persist after hospitalization.

At the same time, our results indicate the significance of handling global patients' conditions, besides the more disease-specific aspects, in those patients with mild or severe neurological status, that may be more prone to poor health quality perception. Furthermore, neurosurgeons should not minimize the impact of these symptoms and inform the patient and caregivers about the risks of these persisting deficits. A detailed explanation of the possibility of long-term incomplete restoration of functional status, along with a worsening of the daily living abilities, should be given to the patients to allow them to deal with them.

It is worth noting that SF-36 mental subscales, such as vitality and social functioning, highlight poor scale scores along with the entire sample, independently from the disability grade, both measured using neurological and self-administered scales. This result could be interpreted as a general inclination to fatigue perception or poor energy, and it would not be surprising to observe this condition in patients who receive a tumor diagnosis and surgical treatments, even a long time after the disease. A preoperative evaluation of patients at risk of worse functional sequelae could be important for tailoring early planning of the best treatment strategies and for an effective rehabilitation program in the postoperative period.

The present study has some limitations. The small sample size and the patients' heterogeneity (e.g., age, histology, and lesion location) reduce its statistical power and limit the possibility to evaluate the impact of other clinical conditions on the patient's long-term wellbeing. It has been demonstrated that postoperative functional outcomes could be affected by tumor histology and location (Raco et al., 2005; Karikari et al., 2011); however, it is not well clear whether those features may induce very long-term dysfunctions. Further investigations need to be conducted in future, extending the statistical analysis to investigate the long-term effects of clinical conditions. Moreover, the retrospective, monocentric nature of this study restricts the cohort of patients to be reached long after the surgery, reducing our sample size. To date, our study was designed to obtain preliminary data allowing physicians to understand patients' needs and in order to turn the spotlight on the patients' long-term QoL in a progression-free survival disease.

## 5. Conclusion

Notwithstanding their greater range of inquiry, the neurological scales do not measure pain, general health, energy levels, emotional functioning, or mental health in patients. Further studies are needed to assess the optimal management of the psychological aspects of spine tumor patients and their surgical outcomes, and our data highlight the long-term impact of neurological decline and the importance of follow-up evaluation after surgery, even with clinical scales that detect everyday patients' needs. Some patients may need closer follow-ups and tailored support to address their QoL decline.

Therefore, it appears crucial to investigate the disease's sequelae, and the overall patients' conditions with a longitudinal multidimensional approach attempting to capture the wide range of symptoms and deficits and minimize the adverse outcomes of IMSCT.

## Data availability statement

The raw data supporting the conclusions of this article will be made available by the authors, without undue reservation.

## Ethics statement

Ethical review and approval were not required for online survey study on human participants in accordance with the local legislation and institutional requirements. Written informed consent was implied via the completion of the questionnaire. All information was treated according to the European regulation GDPR No. 2016/679 with anonymous data sampling.

## Author contributions

EG, MC, and TT: conceptualization, writing—original draft, and writing—review and editing. BC, MM, and TT: data acquisition. ID and GB: data curation. EG: formal analysis and visualization. ID: resources. All authors have read and agreed to the published version of the manuscript.
